# Living in fear at the unpredictability of mental health issues in the classroom: a phenomenological study of secondary school teachers in encountering students with mental health issues

**DOI:** 10.3389/fpsyt.2024.1367660

**Published:** 2024-05-15

**Authors:** Mining Liang, Grace W. K. Ho, Martin Christensen

**Affiliations:** ^1^ School of Nursing, The Hong Kong Polytechnic University, Hong Kong, Hong Kong SAR, China; ^2^ Clinical Nursing Teaching and Research Section, The Second Xiangya Hospital, Central South University, Changsha, China; ^3^ The Interdisciplinary Centre for Qualitative Research, The Hong Kong Polytechnic University, Hong Kong, Hong Kong SAR, China

**Keywords:** secondary school, mental health issues, phenomenology, qualitative study, teachers

## Abstract

**Background:**

The prevalence of mental health issues among secondary school students is on the rise. Secondary school teachers, outside the home environment, are often in a prime position to identify adolescents facing mental health challenges. Limited knowledge regarding the experiences and perspectives of secondary school teachers when encountering this particular group of students, particularly in Asian countries.

**Objectives:**

This study aimed to describe the lived experiences of secondary school teachers exposed to students with mental health issues in the classroom in a Chinese context.

**Methods:**

A descriptive phenomenological approach within the tradition of Husserl was used. A purposive sampling method was used to collect the participants in Changsha, Hunan, China. Sixteen secondary school teachers participated in this study. Individual, face-to-face interviews were conducted, tape-recorded, and transcripted. Colaizzi’s seven-step descriptive phenomenological method was used to do the data analysis.

**Results:**

One Central theme: *Living in fear at the unpredictability of mental health issues in the classroom* and four sub-themes emerged: (1) Worried and anxious by the uncertainty of student mental health issues; (2) Scared and afraid by students’ unpredictable behaviors; (3) Afraid of students’ failure and its potential outcome; (4) Students having mental health issues are dangerous.

**Conclusions and implications:**

The teachers in this study found managing the unpredictability of mental health issues in the classroom deeply distressing and challenging. A comprehensive approach to address the cultural, social, and educational factors influencing secondary school teachers’ experiences is encouraged.

## 1 Introduction

Over the past few years, there has been a growing global concern regarding the increasing prevalence of mental health issues among secondary school students. This rising trend not only has an impact on the academic performance of students but also affects their general well-being and long-term life outcomes (1). Abundant research suggests that approximately 20% of adolescents experience a diagnosable mental disorder, and around 50% of all lifetime cases of mental illness commence before the age of 14 (2). The intricate interaction of biological, psychological, and social factors during the adolescent developmental stage makes teenagers especially susceptible to mental health difficulties. Furthermore, the present-day environment, marked by heightened academic demands, cyberbullying, and societal expectations, has additionally contributed to the escalation of mental health issues among young individuals (3).

Based on Bronfenbrenner’s ecological systems theory, schools (along with other microsystems, including the family) are the most immediate developmental context for adolescents (4). Teachers are expected to play a significant role in school mental health. However, previous studies found that secondary school teachers experience the challenges by observing students with mental health issues (5–7). Secondary school teachers mostly rely on their prior training or judgment to identify students with mental health issues (8). In addition, criteria reported by secondary school teachers in China, such as “tired of learning”, “rebellious”, and “falling in love” at a young age, none of these would be considered to be a symptoms of mental illness by mental health professionals, however, some established signs of mental illness appeared to be normalized by some teachers, such as “self-harm” (9). Indeed, secondary school teachers were just trying their best to identify students with mental health issues despite not knowing what they were doing. Therefore, it is vital to explore their lived experience when encountering adolescents with mental health issues.

Although Western scholars have used a variety of qualitative research methods to examine teachers’ experiences from different perspectives, limited studies have been conducted in the Asian context. The Chinese government has recognized the importance of mental health in schools and has implemented various policies to promote mental health well-being. However, the school mental health system in China faces several challenges: There is often a shortage of trained mental health professionals in schools, the quality and availability of mental health services can vary significantly between different regions and schools. Moreover, as there is a difference in the education system and culture in the Chinese context, the secondary school teachers’ experience of encountering students’ mental health in China could be very different. Therefore, this study aims to understand secondary school teachers’ lived experiences of encountering students with mental health issues in a Chinese context.

## 2 Method

### 2.1 Research question

What is the experience of encountering students with mental health issues in the classroom?

### 2.2 Design

Based on Husserlian descriptive phenomenology (10), this study aims to understand the essence of encountering students with mental health issues based on the lived experience of secondary school teachers. This study was conducted in Changsha City, Hunan, China. China is a country where public education is the mainstay, with approximately 82% of students enrolled in public institutions. Therefore, secondary school teachers interviewed for this study all come from public schools in Changsha City.

### 2.3 Data collection and sampling

To select the participants for this study, purposive sampling was used. The inclusion criterion for this study was individuals who were particularly familiar with or experienced in a relevant phenomenon. In this context, the relevant phenomenon refers to secondary school teachers who had supported students with psychological issues (11). Sixteen secondary school teachers voluntarily participated in the study. Each participant was invited for a 45–60 minute face-to-face interview, which was audio-recorded. Subsequently, the recorded interviews were transcribed verbatim, and the accuracy of the transcripts was verified by comparing them with the original audio recordings. The interview had been completed when data saturation is achieved. Saturation means there was a sufficient understanding of the phenomenon. The interviews were conducted by the first author, who used to work as a psychiatric nurse in a public hospital. The study received ethical approval from the Hong Kong Polytechnic University’s Human Research Ethics Committee (HSEARS20221215002).

### 2.4 Data analysis

The transcripts underwent an initial reading using Colazzi’s seven-step analysis method to gain a comprehensive understanding of the data (12). Phenomenological reduction was employed during this stage to ensure that the author’s personal experiences did not influence the interpretation of the data. Following Whitting’s approach, meaningful units within the transcripts were identified, leading to the development of significant statements (13). These statements were then formulated into meaningful themes, which were further organized into theme clusters. Ultimately, a central theme emerged from the analysis. Throughout this process, reflection was crucial in ensuring that the formulated meanings, sub-themes, and central themes accurately reflected the phenomenon being investigated (refer to **Appendix Table 1** for further details).

## 3 Findings

### 3.1 Participants’ socio-demographic characteristics

The mean age of secondary school teachers was 40.06 years old, and the mean teaching period in secondary school was 16.63 years. There were 11 female teachers and 5 male teachers. The teaching subjects were Math, English, Biology, Physics, Chemistry, Geograph, Computer, Political, etc. (**Appendix Table 2**).

### 3.2 Findings regarding secondary school teachers’ experiences

One central theme was formed: living in fear at the unpredictability of mental health issues in the classroom. Four sub-theme were: “Worried and anxious by the uncertainty of student mental health issues”; “ Scared and afraid by students’ unpredictable behaviors”; “ Afraid of students’ failure and its potential outcome”; “Students having mental health issues are dangerous”.

#### 3.2.1 Worried and anxious by the uncertainty of student mental health issues

The participant highlighted the uncertainty of when these students may face problems. Many of them acknowledged that initially, they may not be aware of the students’ mental health issues. Some of the participants further explained that under normal circumstances, students with mental health issues tended to exhibit reserved behavior in the classroom. The participant described those students as a “transparent” presence in the classroom, meaning that they were easily overlooked or blended into the background. This can be challenging for teachers as they may not be fully aware of the specific challenges these students face or how their mental health issues may manifest. As a result, it became difficult for teachers to anticipate or predict the situations that may arise when these students were dealing with mental health problems. As Guoa and Luo stated:

*You never know when they might encounter problems. Therefore, you must constantly pay attention to them. Initially, we may not be aware that these children have mental health issues. (Guo)*

*In regular circumstances, due to his classroom behavior, he tends to be introverted. However, his behavior does not significantly impact the overall pace of the class or the teacher’s instruction. In the classroom*, he is not the type to actively participate in answering questions, but he also does not disrupt the order of the *class. Therefore, in the classroom, he somewhat resembles a ‘transparent’ presence. (Luo)*

The teacher also expressed surprise and a sense of disbelief because the unwell student did not exhibit any previous indications or signs of mental health issues. They acknowledged that educators may not possess the professional expertise to fully comprehend what was happening inside a student’s mind. Additionally, they noted that another challenging part was that issues could only be identified after they had already happened. As Liao said:

*I was surprised because the student did not show any signs of this situation before. I genuinely thought he was a normal person. Usually, whether he was interacting with teachers, other children, or classmates, he appeared very normal and behaved in a typical manner. (Liao)*

Moreover, the participant expressed a genuine feeling of being overwhelmed and inadequate in handling the situation when students had suicidal thoughts. They mentioned that the suicide attempt signs were extremely subtle, especially given the heavy workload of being a teacher. They felt that the sheer volume of tasks makes it difficult to effectively address the subtle signs exhibited by students. Additionally, they highlighted that students did not directly seek help in an obvious manner but instead hint at their issues indirectly. As a result, the participant found it particularly challenging to navigate the complexities of the situation. As Chen mensioned:

*I genuinely feel that I am unable to handle it because it’s simply impossible to find out, right? Some signs are subtle, especially with the workload of being a head teacher being so heavy. There are various tedious tasks, constant complaints, numerous activities, meetings to attend, and still the need to teach classes. There are just too many things to handle. It is really difficult to effectively address these subtle signs because the students won’t directly seek help in an obvious manner. They will only hint at the issue indirectly. So, it becomes quite challenging, I believe it is quite challenging. (Chen)*

#### 3.2.2 Scared and afraid by students’ unpredictable behaviors

The participants like Hu, expressed her lack of comprehension and confusion regarding self-harm. She stated that she did not understand why this particular emotion and behavior had emerged. She questioned the motive behind self-harm, as she believed that hurting oneself would naturally cause pain. The participant further expressed her inability to grasp why some students would resort to cutting their wrists as a form of self-harm.

*I don’t understand. I don’t know why this emotion arises. I mean, why? Doesn’t hurting oneself cause pain? Why would someone resort to self-harm? I don’t understand why someone would want to cut their wrists. (Hu)*

Xie also described being greatly scared by the severity of the situation when she received a letter written in blood. Xie was concerned about the student’s health and mental state, recognizing that the act of writing such a letter may indicate significant distress and a cry for help. Additionally, the sight of blood and the disturbing nature of the situation evoked feelings of disgust and nausea in Xie. As she said:

*I was scared. There was one time when I received a letter written in blood. She said she wanted to go home and handed it to someone else. When I saw that blood, I couldn’t even dare to open my eyes. It was truly nauseating to see it. (Xie)*

Others like Yang expressed profound shock and disbelief at how the students reached a state where they took their own lives. The participant was left with a deep sense of confusion, questioning what thoughts might have been going through the students’ minds and how they arrived at such a drastic decision. Yang struggled to understand the complex factors that led to such a tragic outcome and the lasting impact it had on them. As he said:

*I am deeply shocked by how this young person ended up in such a state (commit suicide). He had a promising future ahead, yet he didn’t seem to value his own life. How could a person become like this? What thoughts were going through his mind? How did he come to make such a drastic decision? This profound shock lingers in my heart. (Yang)*

#### 3.2.3 Afraid of students’ failure and its potential outcome

Participants believed that mental health issues had an impact on students’ learning abilities. They observed that students with mental health problems tended to be introverted and face challenges in their studies. At the same time, participants agreed with parents’ perspectives that lower expectations for students’ academic performance. Others expressed the belief that the child previously had the potential to be admitted to a more prestigious school, but their mental health issues hindered their academic progress. As Peng stated:

*Because he has mental health issues, his learning abilities are naturally affected. From what I observed, including one very introverted student in the class, his learning abilities have always been in this state since childhood. His parents also don’t have high expectations for him. (Peng)*

Some participants indicated a concern about the student’s ability to cope with adversity and negative feedback. They expressed their observation that the psychological resilience of the students they teach was relatively low. The students tended to attribute setbacks solely to external factors, such as difficult exam questions, instead of their understanding and mastery of the knowledge. The participants further highlighted that these students were more responsive to praise and encouragement, but they found it difficult to effectively handle criticism. Indeed, some participants observed that the students displayed weaker psychological resilience in urban cities and lots of students lacked any significant personal responsibilities for their families’ tasks or chores. As Li said:

*Some students may have difficulty accepting setbacks and failures. I feel that the psychological resilience of the students we teach is quite low, which may be closely related to their experiences in junior high school. They are only receptive to praise and encouragement, but they struggle to handle criticism. If you try to criticize or provide feedback, they tend to have significant stress reactions. (Li)*

Other participants expressed their concern about a prevalent trend within the education system. The participants expressed a sense of frustration and acknowledged the existence of a general atmosphere among educators characterized by fear of potential issues and a reluctance to address students’ weaknesses. This atmosphere resulted in a lack of constructive criticism and hesitancy in providing feedback that could contribute to students’ growth and improvement. The teacher also noted a shift in the perspectives of the student’s parents, who now prioritize encouragement above all else. As Yangmensioned:

*We all have become only focused on discussing the strengths of students and dare not talk about their weaknesses. This situation exists throughout the entire education system, including what I have observed in other schools and our school. It seems that there is a general atmosphere among teachers where they are afraid of potential issues and hesitant to criticize or talk about students’ shortcomings. (Yang)*

#### 3.2.4 Students having mental health issues are dangerous

Some teachers highlighted that when students with mental health issues were faced with triggering events or circumstances, their ability to regulate their emotions becomes greatly compromised. This can manifest as intense emotional outbursts, difficulty managing anger or frustration, feeling overwhelmed by sadness or anxiety, and even self-harm or suicide. Others noted that students facing mental health challenges displayed reluctance to share their problems with others. This hesitance can create a situation where emotions and experiences were suppressed over an extended period, potentially resulting in a dangerous build-up, comparable to a hidden time bomb. Some participants worried about their safety and were afraid that the student might physically assault them or engage in other aggressive behaviors. As Wang and Zhu described:

*Students with mental health issues are similar to normal individuals when nothing has triggered their emotions. However, if something happens that triggers an emotional explosion, their emotional control becomes extremely poor. (Wang)*

*I believe this is a mental health issue. Many people are reluctant to share their mental health problems, experiences, and thoughts with others to have effective communication. As a result, this problem remains like a time bomb. (Zhu)*

The majority of the participants felt they were concerned about the safety of students both during school hours and beyond. For example, some participants mentioned that they had noticed an increase in her phone usage, both at school and at home. On the other hand, Luo also expressed concern about a student’s absence from the classroom and the potential risks or dangers that the student may face outside of the school environment. Some of the participants expressed their genuine fear regarding the possibility of their students engaging in self-harm unexpectedly. Others, like Chen, also spoke of her fear and worry when a student openly expressed thoughts of self-harm or suicide in front of her.

*The first time he disappeared was during our orientation program when he just entered the first year of middle school. He went missing for the whole afternoon, and that was the first time I faced his situation of being absent. At that moment, I felt very worried, and my mind was filled with thoughts of news stories about such incidents. I was afraid that something bad might have happened, and I felt a sense of fear. (Luo)*

*He sat there and said, “The teacher wants me to stay, but I might just jump from here.” He said it right in front of me, and I was quite scared at that moment because I was genuinely afraid that he might be unstable and do such a thing. (Chen)*

Some participants expressed concerns about the potential consequences that may arise if safety issues occur. Participants were genuinely concerned that their reputation and professional image would be damaged as a result. Others recognized that the loss of a student’s life affected the entire school community. Some explained that since they were all located on the same floor if a student from an adjacent class experiences a mental health problem or difficulty, it would inevitably have an impact on the students in the neighboring class. As Peng shared:

*If a serious issue arises, particularly involving personal harm, it would greatly impact me in terms of my reputation. Since I will be staying in this institution for many years, should I prioritize the preservation of my professional image? (Peng)*

*I feel a bit worried myself. I’m afraid that the student might engage in more intense behaviors towards me in the future. Because he is a boy, I perceive him as having significant physical strength and height, which added to my apprehension. I was afraid that he might strike me or engage in other aggressive behaviors towards me. (Peng)*

Having taught students with mental health problems, teachers were inclined to amplify the students’ problems. For example, when they noticed that a student was exhibiting some unusual behavior or phenomena, they became overly concerned about the problem and tended to amplify it. Other participants felt anxious and concerned about the prospect of having a student with mental health issues appear in their classroom again. They anticipated that it could be a challenging experience, causing them distress and making it difficult to manage the student’s behavior or academic performance. As Yang and Liao articulated:

*As soon as you see that the student has some abnormal behavior, you will think in that way, that is, it is easy to expand this matter. It is easy to cause sensitivity and hypersensitivity. (Yang)*

*I don’t know if the student (with mental health issues) will be assigned to my class next semester. If they are, I feel it would still be tormenting. If they are not in my class, perhaps they would be a torment for another teacher. (Liao)*

## 4 Discussion

Mental health issues in the classroom for the secondary school teachers in this study were unpredictable — something they were unaware and students usually did not show any signs. They did not know what signs or symptoms they should be looking for, especially for the signs of attempting suicide. Besides being unaware of the mental health issues, factors such as class size and the subject matter of the course seemed to affect faculty members’ capacity to identify and assist students with mental health illnesses. For instance, due to the class size ranging from 50 to 60 students, participants in our study tend to pay more attention to students at the top, who excel in various aspects such as academic performance, or students at the bottom, who struggle with poor grades and disciplinary issues. However, students who fall in the middle receive comparatively less attention from teachers, making it even more challenging to predict whether this group of students may have mental health issues. The participants in Kalkbrenner’s study also highlighted the challenges of recognizing students facing mental health illness in a large lecture hall (150 students) compared to a smaller classroom setting (10–15 students) (14). Like Buchanan, secondary school teachers decided to assume that every student in the school might be experiencing some form of crisis due to the impossibility of accurately identifying all at-risk students (15). Due to the uncertainty of students’ mental health issues, teachers in our study felt astonished when they aware certain students had mental health issues. Buchanan also found that secondary school teachers expressed shock and surprise upon being informed about the attempted suicide, particularly because they did not expect such an incident to occur and did not know the student even had emotional problems or issues at all (15).

Despite the difficulty in identifying students with mental health issues, teachers were afraid of unwell students’ failure and its potential impact on the classroom. Teachers observed the psychological resilience of the students was relatively low, students tended to attribute setbacks solely to external factors and had difficulty in handling criticism, they behaved indifferently and broke the school regulations and rules without any sense of concern, and they even lacked responsibility for the home chores and tasks in their family, especially for the students in the urban Changsha city. Therefore, teachers concerned about unwell students impacted other students, and worried about their ability to adjust and overcome challenges as they transition into society in the future. There was also a perception that university students today are less resilient compared to previous generations (16). Furthermore, if children’s social, emotional, and behavioral challenges were left unaddressed, it could hindered their ability to learn and thrive academically (17).

Despite their fear of student failure and the adverse effects on the classroom and school environment, teachers harbored fear towards students with mental health issues due to concerns for their safety and the potential negative impact on their professional reputation. On the one hand, they expressed apprehension that the student’s behavior might escalate and become more severe. The teachers were particularly concerned about their own safety and harbored fears of potential physical assault or other aggressive actions from the students. Some college teachers in Allie White’s study also justified their reluctance to initiate conversations about university students’ mental health, as they harbored concerns that such discussions could potentially trigger violent behavior from the students (18). They feared that addressing a university student’s mental health could lead to aggression towards the faculty member, themselves, or others—both during and after the conversation initiated by the teacher (18). On the other hand, teachers expressed apprehension regarding the potential ramifications that could arise from significant incidents, especially those related to students’ safety. Therefore, they were genuinely concerned that their reputation and professional image would be negatively impacted as a result. In addition, three adolescents in Tally Moses’s study also reported that teachers were afraid of adolescents because of their emotional or behavioral volatility (19).

One special facet of this theme, different from the participants in Buchanan’s study, experience with student suicide seemed to make secondary school teachers less shocked and more realistically address the subsequent occurrences of suicide (15). For secondary school teachers in our study, they were concerned about teaching students with mental health issues in the future. The teacher experienced anxiety and apprehension regarding the potential arrival of a new student with mental health issues in their classroom. They anticipated that this situation could present significant challenges, leading to personal distress and making it difficult to effectively handle the student’s behavior and academic performance.

The lived experiences of secondary school teachers encountering students with mental health issues in China were influenced by various cultural, social, and educational factors. In China, there is a social stigma attached to mental health issues, and many teachers fear admitting their struggles due to concerns about professional image and social perception (20). The “zero-COVID” policy in China has also been noted to contribute to increased pressure on teachers and students, impacting mental well-being. Moreover, the traditional exam-oriented education system in some parts of China has been identified as a factor contributing to the mental health challenges faced by both students and teachers (21). On the other hand, the reasons for increasing mental health issues in adolescents in China were exam-oriented education system, parental expectations, and cultural and societal stressors, stigma and misconceptions, which were different from Western countries, such as social media and technology, adverse childhood experiences, access to mental health services (22, 23). Therefore, those differences highlight the need for a comprehensive approach to address the cultural, social, and educational factors influencing secondary school teachers’ experiences in facing students with mental health issues. Our study provide the evidence for future study and interventions.

### 4.1 Implications

The secondary school teachers in this study found managing the unpredictability of mental health issues in the classroom deeply distressing and challenging. The worry about student safety calls for a dual approach: improving teacher preparedness through specific training in crisis management and enhancing proactive measures within schools to identify and support students at risk before crises occur. Such training should include simulation-based learning, which could help reduce the shock factor by providing realistic scenarios for teachers to engage with in a controlled environment. Future research should explore the effectiveness of proactive mental health programs in reducing both student incidents and teacher anxiety.

### 4.2 Strength and limitations

There are some limitations to interpreting our study findings. The secondary school teachers all came from Changsha City in China, making the findings inapplicable to secondary school teachers in other cultures. However, the strength is that our study is the first study to use a descriptive phenomenological design to explore the lived experience of secondary school teachers in encountering students with mental health issues in a Chinese context.

## 5 Conclusion

The participants in our study described their feelings of uncertainty about whether students had mental health issues and found it challenging to identify students who may have such issues, leading to the potential overlooking of students with mental health problems. They also notice a decreased psychological resilience among current students. Over time, teachers begin to develop an understanding of students’ mental health issues and their impact on the classroom, they grow increasingly concerned that mental health issues as dangerous. Our recommendation is for adolescents to enroll in at least one course per semester that has a small class size, typically consisting of approximately 25 students or fewer. This approach can foster more frequent interactions between secondary school teachers and students. Additionally, the finding of our study that teachers’ experience of living in fear when encountering students with mental health issues encourages secondary school nurses to develop questionnaires and interventions for future studies.

## Data availability statement

The original contributions presented in the study are included in the article/supplementary material. Further inquiries can be directed to the corresponding author.

## Ethics statement

The studies involving humans were approved by Hong Kong Polytechnic University’s Human Research Ethics Committee. The studies were conducted in accordance with the local legislation and institutional requirements. The participants provided their written informed consent to participate in this study.

## Author contributions

ML: Conceptualization, Data curation, Formal analysis, Investigation, Methodology, Writing – original draft, Writing – review & editing. GH: Conceptualization, Supervision, Writing – review & editing. MC: Conceptualization, Formal analysis, Methodology, Supervision, Validation, Writing – review & editing.

## Appendix

**Table 1:** Living in fear at the unpredictability of mental illness in the Classroom – From Formulated Meaning to Central Theme.

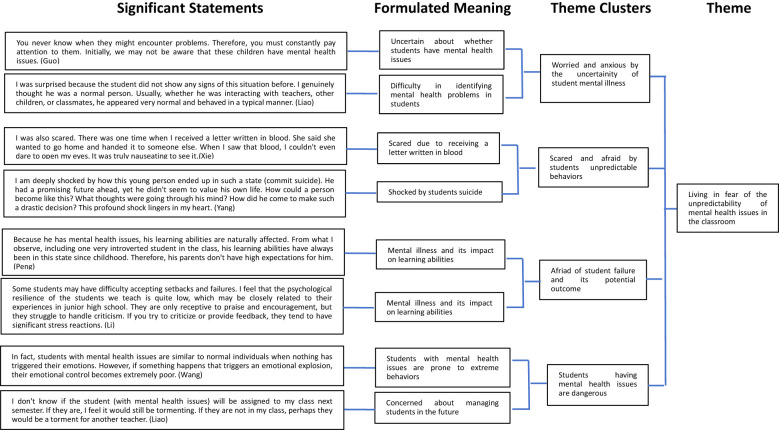

**Table 2:** The demography of the secondary school teachers participated in the interviews.

Name	Age	Academic background	Duration of teaching	Teaching courses
Zhu	47	Undergraduate	20	English
Peng	33	Master’s degree	6	Political Ideology
Hu	48	Undergraduate	23	Computer
Han	48	Undergraduate	25	Geography
Wang	53	Undergraduate	35	English
Yang	43	Master’s degree	17	Mathematics
Wang	32	Master’s degree	10	Chinese
Luo	25	Master’s degree	2	Mathematics
Chen	26	Master’s degree	1	Biology
Guo	55	Undergraduate	37	Mathematics
Li	37	Undergraduate	13	English
Liao	44	Master’s degree	15	physics
Xie	26	Master’s degree	3	English
Wu	42	Undergraduate	20	Chemistry
Liu	30	Master’s degree	9	Chemistry
Nie	52	Undergraduate	30	Geography
